# Efficient patient-initiated follow-up after orthopaedic injections

**DOI:** 10.1007/s00590-025-04193-9

**Published:** 2025-02-25

**Authors:** Ka Siu Fan, Iqraa Haq, Ali Taha, Andrew Carne, Chintu Gademsetty, Matthew Solan

**Affiliations:** 1 Royal Surrey County Hospital, Guildford, UK; 2 University of Surrey, Guildford, UK

**Keywords:** Patient-initiated follow-up (PIFU), Pain diary, Foot, Ankle, Injections, Appointment

## Abstract

**Background:**

Annually, thousands of patients with foot and ankle pain receive injections for both diagnostic and therapeutic purposes. However, since the duration of pain relief is unpredictable, planning follow-up appointments is difficult. This study aimed to improve patient-initiated follow-up through pain diaries.

**Methods:**

The diagnosis, intervention and outcomes for patients receiving ultrasound guided foot and ankle injections were included. By comparing the timing of patient-initiated appointments against the standard 6–8 week appointments, the number of ‘saved’ appointments were calculated.

**Results:**

Over twelve months, 104 injections were recorded. Fifty-nine patients (57%) requested a follow-up, with a median time to follow-up was 117 days. Only nine patients (9%) requested a review within 6–8 weeks. This may translate to a potential saving of £9500–£19,000 from the avoided follow-up appointments.

**Conclusion:**

Improved pain monitoring through pain diaries allows follow-up appointments to be tailored to patients, which can improve patient experience and resource allocation.

## Introduction

Image-guided foot and ankle injections are commonly used as a diagnostic and/or therapeutic procedure for pathology such as tendinopathy or arthritis [1]**.** These injections are often performed by orthopaedic surgeons or radiologists under ultrasound imaging, to deliver corticosteroids and local anaesthetic directly into the site of suspected pathology. The extent to which the initial effect (local anaesthetic) of the injection alleviates pain serves as a diagnostic tool to localise the pain and confirm the clinical diagnosis [2, 3]. The corticosteroid in the injection is for longer-lasting pain relief. The duration of pain relief is highly variable [4]. Monitoring of pain after the injection can help guide the timing of further intervention. The best time for a follow-up appointment after an injection is difficult to predict. There is no ‘gold-standard’ time for follow-up and clinicians often aim to review patients after 6–8 weeks.

Options include:

1. Routine appointment: A 6–8 week appointment is a common choice. A recent questionnaire survey found that 85% of clinicians routinely review their patients 6–8 weeks postinjection [5]. In most cases, the injection was still effective and so no useful management decision was made at this 6–8 week appointment.
2. Discharge to GP: In the same survey, a minority of clinicians chose to discharge the patient after injection. This is, arguably, not constructive in a system, where re-referral to the secondary care team is bureaucratic and time-consuming.
3. Later follow-up: An appointment at a later time, particularly if the injection has worn off, makes the immediate (diagnostic) effect of the local anaesthetic difficult for the patient to recall. Important clinical information is lost. The initial degree of benefit from the injection is useful information and helps determine whether the patient would benefit from additional treatment or whether alternative diagnoses need to be considered instead [6, 7].

In the context of a strained healthcare system and the ever-increasing backlog of outpatient appointments, it is important to identify individual patient need in order to allocate resources effectively [8]. Given the variability of patient presentation, underlying pathology and the duration of postinjection pain relief, routine follow-up appointments may not always be required or appropriately timed. A recent survey identified that 83% of routine appointments were unnecessary [5]. Patients were brought back for review too soon, at a time when they did not require further help [9].

An increasingly popular initiative called Patient-Initiated Follow-Up (PIFU) streamlines resources, empowering patients to seek care as and when they feel it is needed [10]. PIFU can be used in place of a scheduled routine follow-up appointment. This encourages patients to be more involved in their own care and pain management, whilst also ensuring appointments are made only when required. Benefits include improved pathway efficiency, financial savings, better patient experience and reduced waiting times [11].

There is an increased push towards personalised, patient-centred care within healthcare systems to enable patients to make informed decisions about their health. Monitoring their pain scores can guide the decision to initiate follow-up [12]. We have used a Pain Diary to track pain levels after foot and ankle injections. Based upon the individual response to injection arrangements for timely follow-up are then made.

This study aimed to quantify the savings made through use of a Pain Diary to support personalised follow-up.

## Methods

All patients who underwent injection of the foot and ankle under the care of a single consultant at a district general hospital were included. Electronic patient notes, clinic letters, and radiological procedure records were examined. Information including patient age, indication for injection, anatomical site, findings of the preliminary USS, subsequent management, outcomes, and follow-up attendance were recorded.

**Fig. 1:**
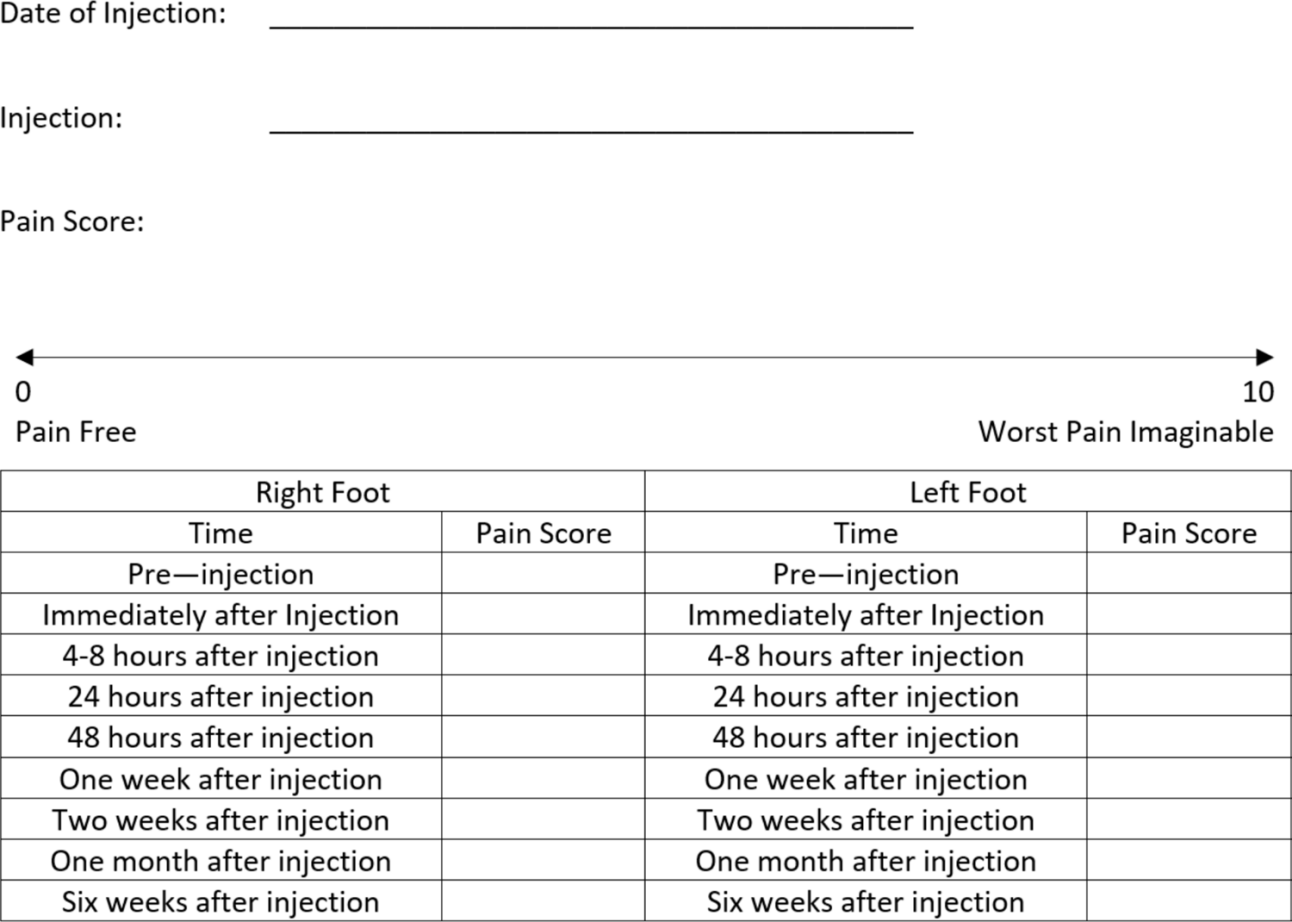
Pain diary provided to patients to capture the pain levels and location of pain throughout the weeks following their injection

The Pain Diary was provided to the patient as a paper leaflet at the end of their outpatient consultation or (if mislaid) at the time of the injection. The Diary used a structured table to record pain levels on a visuo-analogue scale (Fig. 1). Patients were given instructions to return the Diary after six weeks. Based on the pain levels recorded in their diary, patients were either offered a review appointment or provided with details about how to refer themselves back later if necessary (PIFU). By comparing these outcomes with a standard 6–8 week appointment, the number of saved appointments was calculated. While the costs of orthopaedic appointments can vary between location, trust, and type of appointment, the potential cost savings were calculated using a range of £100–200 per follow-up appointment from existing literature [13, 14].

This study was conducted with the approval from the Audit/Quality Improvement team at Royal Surrey County Hospital, Guildford, Surrey, GU2 7XX, United Kingdom (Audit reference SU-CA-22–23-058).

## Results

A total of 115 foot and ankle ultrasound guided injection referrals were identified. All were from a single surgeon’s team over a 12-month period. Fifty-two foot, 29 ankle, and 34 Achilles ultrasound scans were performed. One hundred and four (104) patients received injections. One injection was performed by the orthopaedic surgeon under fluoroscopy, the rest were performed by a radiologist under ultrasound guidance.

Eleven patients had no injection: three patients declined injections and eight were no longer clinically indicated. These patients reported that their pain had improved, while awaiting the injection appointment. The time between injection and subsequent follow-up ranged from seven to 484 days, with a median of 117 days.

Of the 104 injections, only 59 (57%) requested a follow-up appointment, of which only 9% would have benefitted from a standard 6–8 week appointment (Table 1). Forty-three percent of patients requested no follow-up at all. Some of these patients may return for further care later. Of the patients who requested a review appointment, the majority did so after more than three months postinjection. Only 9% of all patients requested review within the 6–8 week period. Ninety-one percent of the patients had no need for a routine 6–8 week follow-up, which may be extrapolated to a potential saving of £9500 to £19,000 from the avoided follow-up appointments alone (Table 2).

**Table 1:** Time period when follow-up requested by the 57% of patients who required review

Follow-up time	Number of patients (%)
At or before 6–8 weeks	9 (9%)
Within 90 days	11 (11%)
> 90 days (3–18 months)	39 (38%)

**Table 2:** Extrapolated cost savings based on the reported estimates of appointment costs

Approach	Initial appointment attendance (*n*)	Initial appointment costs	6–8 Week follow ups (*n*)	Follow up appointment costs	Total costs
Standard 6–8 Week Appointment	104	£200	104	£100–£200	£31,200–£41,600
With PIFU			9		£21,700–£ 22,600

## Discussion

Corticosteroid with local anaesthetic injections are a widely utilised diagnostic and therapeutic tool. The local anaesthetic provides immediate pain relief at the anatomic site injected and this helps to confirm the clinical diagnosis. For ongoing pain management, the corticosteroid reduces local inflammation. However, the duration of pain relief from injections varies significantly [3, 10]. Without any reliable means to predict the post-intervention outcome, there are no guidelines for standardised follow-up timing. The results of our study support a more patient-centred pathway that relies on self-reported pain levels to determine when follow-up appointments are required.

This study reviewed all the patients referred for foot and ankle injections from a single consultant's team at a district general hospital over one year. By using pain diaries, we were able to identify the actual needs of patients following the intervention. Our findings demonstrate that only a minority of patients (9%) required a follow-up appointment within 6–8 weeks. This means that the widely accepted ‘standard’ 6–8 week appointment is unnecessary for most patients. Using the costs of the initial clinic assessment and follow-up appointment, the potential overall costs was calculated from the studied patient cohort. While a range £100–200 per appointment was used to reflect the variation in base cost of appointments, the potential saving may be as high as £9500 to £19,000 for approximately 100 patients alone. As this only includes the avoided follow-up appointments, this may translate to much greater savings when implemented across all orthopaedic steroid injection appointments and at large NHS trusts. By moving away from traditional appointment timeframes to a more autonomous and patient-centred approach, healthcare resources can be used more effectively [8].

Using pain diaries with predetermined times for pain score recording provides valuable clinical information. A detailed record informs future decision-making and aids shared decision-making with regards to, for example, a repeat injection at the same site, or perhaps (if the injection provided no initial relief of pain) injecting a neighbouring anatomic location instead [15]. For pain that improves with injection, but the relief does not last, alternative surgical interventions such as joint fusion may be considered. Confidence that such surgical treatment will be effective is much greater after an injection has proven to provide temporary relief. Confidence that the source of pain has been correctly identified is improved by the patient’s use of a pain diary [16].

Limitations with this pathway of care fall into two categories. Firstly, the diligent use and accuracy of pain diaries. Success relies on patients regularly and consistently recording their pain levels. Paper forms can be lost or completed at the last minute instead of at regular intervals, thereby failing to capture the information reliably.

Secondly, there are drawbacks associated with PIFU. Obstacles to its implementation include cultural change; patient engagement; and the risk of losing patients (especially the more vulnerable ones) to follow-up. Another potential issue when promoting this pathway as a means of saving appointments is the ‘paradoxical’ increase in hospital outpatient appointments. In a mixed-methods evaluation, funded by the National Institute for Health and Care Research, the use of PIFU in 29 specialities saw a rise in outpatient attendances in seven specialities, perhaps due to improved accessibility [17].

To overcome problems associated with a paper diary system we are now using a digital Pain Diary in a pilot study. This may provide even more detailed and real-time patterns of data to support patient care [18]. The documented pain history may also be shared within the clinical team to facilitate consultations if patients cannot see their usual clinician. Those patients repeatedly failing to complete their scores can be contacted directly so that the risk of vulnerable patients slipping through the net is minimised. Other benefits of a digital pain diary include remote monitoring of scores by clinicians who can then initiate consultations, or a repeat injection, when pain score rises.

This study was only for injections to the foot and ankle. Injections are widely used in all other orthopaedic subspecialities, pain clinics and rheumatology. These clinical services are all under huge pressure to see more patients. If a single foot and ankle consultant’s team can save nearly 100 appointments a year, the potential for savings across the NHS is enormous.

## Conclusion

Injection of local anaesthetic and corticosteroid is a common procedure for patients with foot and ankle pain. A routine follow-up 6–8 weeks after the injection is not appropriate for patients who remain asymptomatic. Our findings provide evidence to support the use of a pain diary to underpin a patient-initiated follow-up pathway instead. This provides both an improved patient experience and financial savings for the healthcare system. Future work, using digital pain diaries, is likely to bring further improvements in efficiency.

## Data Availability

This study was conducted with the approval from the Audit/Quality Improvement team at Royal Surrey County Hospital, Guildford, Surrey, GU2 7XX, United Kingdom (Audit reference SU-CA-22-23-058). Anonymised data may be made available upon reasonable request.
